# Rapid and Sensitive Assay of *Helicobacter pylori* With One-Tube RPA-CRISPR/Cas12 by Portable Array Detector for Visible Analysis of Thermostatic Nucleic Acid Amplification

**DOI:** 10.3389/fmicb.2022.858247

**Published:** 2022-05-02

**Authors:** Bing Dai, An Xiang, Di Qu, Guo Chen, Li Wang, Wenwen Wang, Dongsheng Zhai, Lei Wang, Zifan Lu

**Affiliations:** ^1^The College of Life Sciences, Northwest University, Xi’an, China; ^2^Department of Biopharmaceutics, State Key Laboratory of Cancer Biology, Air Force Medical University, Xi’an, China; ^3^Department of Gastrointestinal Surgery, General Hospital of Ningxia Medical University, Ningxia, China

**Keywords:** *Helicobacter pylori*, CRISPR/Cas12a, recombinase polymerase amplification, *vacA*, *cagA*, *16SrDNA*, naked eye detection

## Abstract

*Helicobacter pylori* (*H. pylori*) has infected more than half of the world’s population and is still a threat to human health. The urea breath test, despite being widely used in clinical diagnosis, still faces huge challenges in the immediate detection of *H. pylori*. Thus, a rapid, sensitive, and highly specific point of care diagnosis is particularly important for preventing the further transmission of *H. pylori* and for real-time monitoring of the disease in a given population. Recently, the clustered regularly interspaced short palindromic repeats (CRISPR)-based diagnostics have been applied to various types of nucleic acid testing; however, there are often shortcomings of complex operation and high signal transmission background. In this study, we proposed a new platform for the assay of *H. pylori* using one-tube-based CRISPR/Cas12a diagnostic methods and designed a detector for this platform, which is a portable array detector for visible analysis of thermostatic nucleic acid amplification (Pad-VATA). By incorporating isothermal recombinase polymerase amplification, our platform could detect the conserved gene fragments of *H. pylori* with a constant low as 2 copies/μl. The assay process can be performed at a single temperature in about 30 min and integrated into the reactor in the palm-sized Pad-VATA to facilitate rapid diagnosis of *H. pylori*. We also verified the accuracy of our platform using 10 clinical samples and found that the platform can quickly detect *H. pylori* infection in a given population. We believe that this fast, convenient, efficient, and inexpensive screening and diagnostic platform can be widely used in various settings, including homes and clinics.

## Introduction

*Helicobacter pylori* (*H. pylori*) is a Gram-negative, microaerobic pathogen that generally colonizes the human gastric mucosa tissue and causes diseases such as gastritis, peptic ulcers, stomach cancer, and gastric mucosa-related lymphoma (Plummer et al., 2015; Wang et al., 2015; Eisdorfer et al., 2018). More than half of the world’s population is infected with *H. pylori* (Eusebi et al., 2014). Therefore, it is listed as the first class of biological carcinogens by the World Health Organization (WTO) (Hooi et al., 2017). At present, management of the epidemic infectious disease caused by *H. pylori* still relies on rapid and accurate diagnosis (Hatakeyama, 2014; Hooi et al., 2017; Liu et al., 2018). In recent years, although several monitoring *H. pylori* have been advanced greatly, some limitations still exist: (1) Gastroscope biopsy method, with low detection specificity, high cost, and the need for professional operators and large-scale testing equipment. Moreover, it is easy to cause secondary trauma to patients owing to the invasive examination (Cho et al., 2013; Kato et al., 2013; Pohl et al., 2019). (2) Blood test, which is designed to test the levels of *H. pylori* antibody in the serum to determine whether there is *H. pylori* infection. The disadvantage of this approach is that the false-negative results when the test is done in the early phase of infection, thus depriving the patient of the best time for treatment. The other circumstance is the false positive. For example, there may still be some antibodies in the blood after the patient has been recovered (Malfertheiner et al., 2012; Yang et al., 2015). (3) Urea breath test (UBT), through ^13^C or ^14^C isotope marker detection of *H. pylori* by *H. pylori* testing, requires stopping 2 weeks of proton pump inhibitors (PPIS), such as rabeprazole, omeprazole, pantoprazole, and lansoprazole, and 4 weeks of antibacterial drugs, such as amoxicillin, and kanamycin; otherwise, it will result in false-negative results (Velayos et al., 2012; Wang et al., 2015). Recently, molecular diagnostic techniques have been increasingly applied in *H. pylori* monitoring. *Helicobacter pylori* can be detected by polymerase chain reaction (PCR) using gastric biopsy specimens (Wang et al., 2015), saliva, feces, and gastric juice (Momtaz et al., 2012; Saez et al., 2012), among which the results of bleeding patients are more accurate. Although PCR has good accuracy and sensitivity for *H. pylori* monitoring, it still relies on large-scale experimental equipment and professional operators and cannot meet the requirements of rapid and instant bedside detection in practical applications. Therefore, developing a high-specificity, high-sensitivity, low-cost, fast, and portable method for *H. pylori* detection will largely benefit the patients and clinicians in making a quick diagnosis to ensure timely intervention and treatment of *H. pylori* and prevent the widespread of this infectious disease.

Recently, clustered regularly interspaced short palindromic repeats (CRISPR) associated (Cas), CRISPR/Cas protein-based nucleic acid detection technology has been gradually applied to detect various targets (Terns and Terns, 2011; Pawluk et al., 2018; An et al., 2021), and its specificity and sensitivity are good, compared with PCR technology. Meanwhile, the technique combined with isothermal amplification (Gootenberg et al., 2017; Li et al., 2018) can achieve instant detection of the point of care testing and show a good future in molecular diagnosis (Wang et al., 2020; Xiong et al., 2020) and a good prospect in the molecular segment (Dai et al., 2019; Hou et al., 2020; Lee et al., 2021; Kashefi-Kheyrabadi et al., 2022). The CRISPR/Cas12a protein was previously known as Cpf1, and it is a pronuclear deoxyribonuclease that can be programmed through guide RNA to target complementary DNA sequences. Then, Cas12a specifically cuts DNA (called cis cleavage), requiring the assistance of the PAM sequence. Particularly, Cas12a was able to form a ternary complex with crRNA and target DNA, showing the strong “random cutting” activity and cutting any single strand of DNA in the system into fragments (called trans cleavage) (Garcia-Doval and Jinek, 2017; Wang et al., 2021). (Chen et al., 2018) innovatively combined cas12a-targeted cutting single-stranded DNA and non-targeted cutting arbitrary single-stranded DNA with recombinase polymerase amplification (RPA) technology to develop a technique named DETECTR (DNA Endonuclease-Targeted CRISPR Trans Reporter). The anal swab sample is isothermally amplified for 10 min, the amplification products are detected using the CRISPR/Cas12a system, and two similar subtypes of human papillomavirus 16 (HPV 16) and HPV 18 can be detected and accurately distinguished within 1 h (Chen et al., 2018). Another study proposed a new version of “SHERLOCK” (Gootenberg et al., 2017), which combines Cas13 and test strips more quickly and accurately to detect genetic features in samples, including pathogen and tumor DNA, through a miniature paper test, and even to observe its results with the naked eye. However, the amplification of the target and the downstream Cas signal amplification processes are still separated in the two containers in these pioneering platforms, which result in the risk of open lid contamination. At the same time, the fluorescent signal from the fluorophore burst is still monitored by large equipment, and thus, the entire operation process is complicated.

In this study, we constructed a newly rapid *H. pylori* detection platform, and the results obtained from that can be easily identified by the naked eye. The new platform combined CRISPR/Cas12a with RPA and achieved an integrated reaction through the internal space allocation of the test tube and the optimization of the monitoring process, eliminating the need for open lid operation and avoiding false-positive results caused by aerosol contamination. Based on this approach, we have designed a portable array detector for visible analysis of thermostatic nucleic acid amplification (Pad-VATA) that monitors the fluorescence signal throughout the process without the need for complex operational procedures. The real-time, high-sensitivity fluorescent readout-based RPA-CRISPR assay can achieve *H. pylori* detection with a limit of detection of 2 copies/μl within 30 min. Furthermore, it was easy to be operated and could be used in home, field, battlefield, and other sites to achieve initial screening and diagnosis of *H. pylori*.

## Materials and Methods

### Materials and Reagents

All reactions involved primers, ssDNA-reporter (FAM-TTATT-BHQ1), and CRISPR RNA (crRNA) synthesized by Sangon Biotech (Shanghai, China). EngenLba Cas12a, nuclease-free water, and 10 × NEbuffer were purchased from New England Biolabs (Ipswich, MA, United States). The TwistAmp Basic kit (#TABAS03KIT) for RPA was purchased from TwistD× Limited (Maidenhead, United Kingdom). The PCR Master Mix was purchased from Takara (Dalian, China). The Quantitative Real-time PCR (qPCR) Master Mix was purchased from YEASEN (Shanghai, China). The pUC57 recombinant plasmids containing *vacA*, *cagA*, and *16SrDNA* genes of *H. pylori* were transformed by *E. coli*, and the above process was done by Sangon Biotech (Shanghai, China). Genomic DNA Extraction Kits were purchased from TIANGEN Biotech (Beijing, China).

Clinical saliva samples were provided by the Tang du Hospital of Air Force Military Medical University (Xi’an, China). The study was approved by the Air Force Military Medical University Research Ethics Review Board. A written informed (verbal) consent was obtained from all patients. Saliva samples were collected from all volunteers after undergoing ^13^C-UBT.

### Instrumentation

All incubations in the preliminary experiments of this study were performed on a metal bath; all real-time fluorescence signals were monitored by Roche Light Cycler^®^ 480 Real-Time Fluorescence detection system. Every 45 s, fluorescence signals were collected and reflected in the form of a curve. The endpoint visual fluorescence signals were detected with a blue light transilluminator and recorded using a cell phone photo. The incubation process and the visual fluorescence signal of subsequent experiments were based on a Pad-VATA; the fluorescence signal was then recorded using a cell phone photo.

The Pad-VATA is composed of temperature-controlled components and ultraviolet (UV) detection components. Specifically, it is mainly composed of UV lamp columns, filter plates, temperature control components, and aluminum baffles. The temperature control unit comprises four groups of aluminum sample carriers with 64 holes that are suitable for PCR strip tubes (volume: 200 μl). The sides of each aluminum part have a piece of polyimide heating film (12 V, 5 W), which also includes a digital temperature controller with an accuracy of 0.1°C and a temperature probe with a refresh frequency of 0.5 s. The UV lamp array comprises 64 LED light beads (365 nm, 12 V, 5 W), with each located directly below the sample tube. The aluminum baffle on the cover has the same hole array as the sample tube and has a contact area with the temperature-controlled aluminum for maintaining the temperature of the sample tube cover and eliminating the lamp interference at the edge of the detection hole.

### Amplification of Target Fragments

For PCR, 1 μl of DNA template was mixed in 49 μl of PCR mix containing 25 μl of 2 × Taq Master Mix, 1 μl of forward primer (10 μM), 1 μl of reverse primer (10 μM), and 2 μl of nuclease-free water. The DNA amplification protocol was as follows: 95°C for 5 min, 40 cycles at 95°C for 30 s, 60°C for 30 s, 72°C for 30 s, and 72°C for 5 min. Finally, 20 μl of the amplification product was subjected to agarose gel electrophoresis.

For qPCR, 1 μl of DNA template was mixed in 9 μl of PCR mixture containing 5 μl of 2 × Taq Master Mix, 0.5 μl of forward primer (10 μM), 0.5 μl of reverse primer (10 μM), and 3 μl of nuclease-free water. The DNA amplification protocol was as follows: 95°C for 5 min, 45 cycles at 95°C for 10 s, and 60°C for 35 s (fluorescence signals were measured at this step). Afterward, a melting temperature curve analysis was performed.

For RPA, RPA pellets were resuspended in 29.5 μl of the supplied rehydration buffer containing 2.4 μl of forward primer (10 μM), 2.4 μl of reverse primer (10 μM), and 12.2 μl of nuclease-free water. Subsequently, 2.5 μl of MgOAC solution and 1 μl of template were added to a well-mixed RPA reagent and incubated at 37°C for 20 min. The amplified product was subjected to agarose gel electrophoresis and used for subsequent experiments.

### *Helicobacter pylori*-Specific CRISPR RNA Design

To achieve a high detection sensitivity, specific crRNAs for different domains of *vacA*, *cagA*, and *16SrDNA* genes were designed using the CRISPR-DT software.^1^ To assess the efficiency of crRNA by enabling crRNA to target specific TTTN PAM sequences, we screened out the highest-scoring sequences through software comparison.

### Optimization of the CRISPR/Cas12a-Based Fluorescence Detection System

CRISPR/Cas12a lysis reactions were performed as follows: all reaction processes are performed in EP tubes, 3 μl of the RPA product was added to 28.5 μl of the CRISPR mixed reaction system containing 1.5 μl of Cas12a (1 μM), 1.5 μl of crRNA (1 μM), 1.5 μl of ssDNA-reporter (10 μM), 3 μl of 10 × NEBuffer 2.1, and 21 μl of nuclease-free water. Afterward, the above reaction setup was well mixed and incubated at 37°C for 20 min, and the Light Cycler 480 fluorescence detection system was used to detect the fluorescent signal.

For substrate dynamics studies that rely on Cas12a, the concentrations of crRNA were set to 12.5, 25, 50, 75, and 100 nM for the analysis. The concentrations of ssDNA-reporter were set to 125, 250, 500, 750, 1,000, 1,500, and 2,000 nM for the analysis.

### A One-Tube Fluorescence Visual Detection Assay

For this assay, 30 μl of the one-tube CRISPR/Cas12a lysis reaction mixture containing 1.5 μl of Cas12a (1 μM), 1.5 μl of crRNA (1 μM), 1.5 μl of ssDNA-reporter (20 μM), 3 μl of 10 × NEBuffer 2.1, and 21 μl of nuclease-free water were added to the bottom of the tube. Afterward, 10 μl of the RPA unreacted mixture containing 5.9 μl of 2 × reaction buffer, 0.5 μl of forward primer (10 μM), 0.5 μl of reverse primer (10 μM), 2.4 μl of nuclease-free water, 0.5 μl of MgOAC solution, and 1 μl of DNA template was added inside the tube’s lid. The tubes were then covered and placed in a 37°C thermostatic reactor for 20 min, the amplification products inside the tube’s lid were immediately flung to the bottom of the tube and mixed well with the CRISPR/Cas12a system, followed by naked eye monitoring of the fluorescence signal under UV light.

### Mimicked Clinical Samples and Clinical Samples Analyzes

Five saliva samples from healthy individuals were randomly obtained with a saliva collector and properly mixed and added with gradient dilutions of plasmids of different copy numbers as positive templates. Pure saliva samples without plasmids served as negative controls. The material was boiled in boiling water (100°C) for 10 min and used as a mimicked clinical sample for subsequent CRISPR testing. The diluted plasmids were used as templates for the qPCR investigations.

Ten clinical saliva samples were collected with a saliva collector and processed in boiling water for 10 min to lyse the nucleic acids. The processed samples were then used for subsequent experimental analyzes.

## Results

### Principles of *Helicobacter pylori* Detection

**FIGURE 1:**
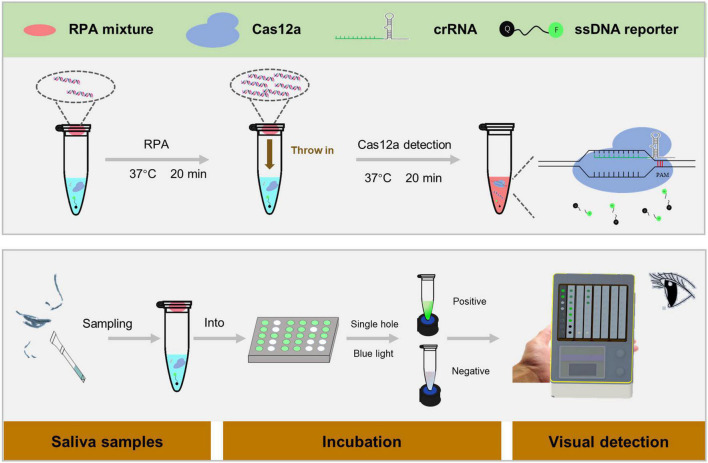
Scheme of the one-tube RPA-CRISPR assay platform for *Helicobacter pylori* detection with the naked eye. Within 5 min, the saliva sample was processed in boiling water to obtain DNA, and 1 μl of the treated sample was added to the RPA mixture system for CRISPR detection of *H. pylori*. The specific protocol is as follows: 30 μl of CRISPR/Cas12a reaction mixture is added to the bottom of the tube, and 9 μl of RPA unreacted mixture and 1 μl of the sample are added inside the tube’s lid, which is placed on Pad-VATA for incubation. After 20 min incubation at 37°C, the amplified DNA sample inside the tube’s lid is mixed with the CRISPR reaction solution at the bottom of the tube by shaking the tube. The trans-cleavage activity is instantly activated to cut any ssDNA-reporter once the Cas12a enzyme is specifically identified with the target DNA; meanwhile, the fluorescent signal can be recognized by the naked eye.

Amplification of the target fragment and trans cleavage experiments of Cas12a were incorporated into a one-tube RPA-CRISPR assay platform for the detection of *H. pylori*. Specifically, crRNA, Cas12a, and target DNA fragments amplified by RPA form a ternary complex. The *H. pylori* DNA fragment is then specifically cleaved by Cas12a following the activation of the cis-cleavage activity of Cas12a. Eventually, the trans cleavage activity of Cas12a is also activated, which leads to a random cleavage of the ssDNA-reporter by Cas12a and the generation of a strong fluorescent signal. All reactions were performed on our specially constructed Pad-VATA (Figure 1).

### Establishment and Optimization of a Positive Amplification System for *Helicobacter pylori* Detection

**FIGURE 2:**
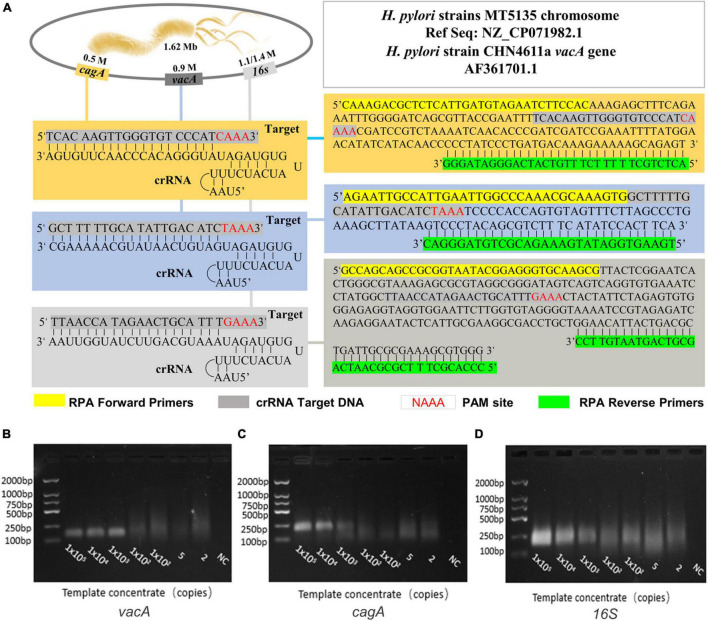
The establishment and optimization of a positive amplification system for *H. pylori* detection. **(A)** The genome map shows the target site of the *H. pylori vacA*, *cagA*, and *16SrDNA*, and it also shows the sequence of crRNA and the location of the RPA primer sequence in this assay. The RPA amplification products **(B)** *vacA*, **(C)** *cagA*, and **(D)** *16S* were analyzed by agarose gel electrophoresis with the dilution of DNA as a template during amplification.

We first compared the sequence of the *H. pylori* polymerase encoding area, screened out the conservative sequence that matched the Cas12a original interval proximity sequence (5-TTTN), and synthesized the plasmids as a positive template for subsequent experimental design. Based on this target area, the crRNA and RPA primers of *H. pylori* are shown in Figure 2A and Supplementary Table 1. These recombinant plasmids were continuously diluted with different gradients for RPA, followed by agarose gel electrophoresis. The results showed that the *H. pylori*-positive detection system was successfully constructed (Figures 2B–D).

### Construction and Optimization of the RPA-CRISPR Method for *Helicobacter pylori*

**FIGURE 3:**
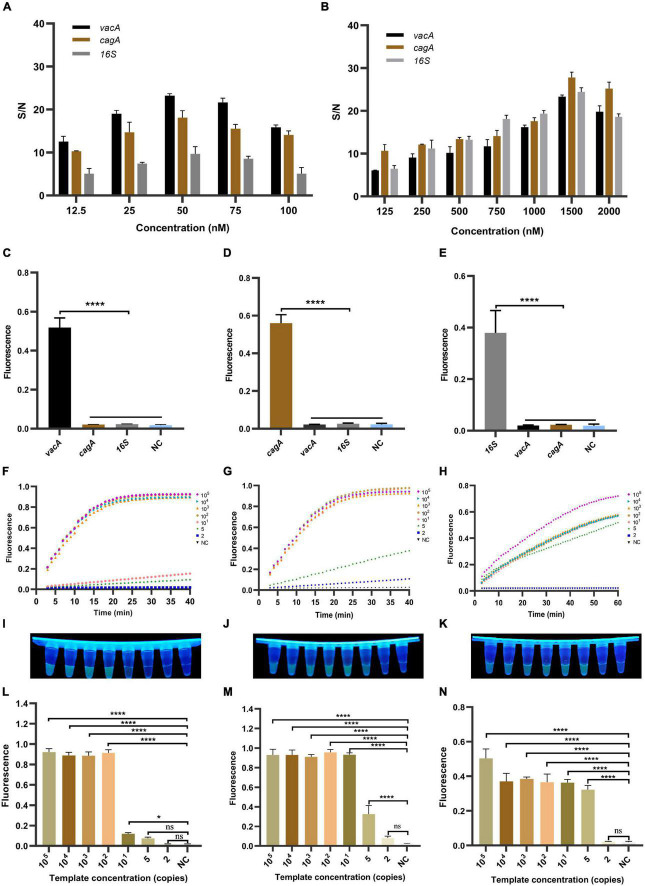
The establishment and optimization of the RPA-CRISPR method for *H. pylori*. RPA-CRISPR method signals depend on the concentration of **(A)** crRNA and **(B)** ssDNA-reporter. For the selectivity of **(C)** *vacA*, **(D)** *cagA*, and **(E)** *16SrDNA*, the positive samples were all diluted templates (10^5^ copies/μl). The fluorescence curves of **(F)** *vacA*, **(G)** *cagA*, and **(H)** *16SrDNA* generated by the Light Cycler 480 fluorescence detection system at each dilution within 30 min. The fluorescence intensity values with diluted templates of **(I)** *vacA*, **(J)** *cagA*, and **(K)** *16SrDNA* are detected by UV device at 30 min, and the corresponding fluorescence values with diluted templates of **(L)** *vacA*, **(M)** *cagA*, and **(N)** *16SrDNA* are quantified by the Light Cycler 480 fluorescence detection system at 30 min. Negative controls (NC) were replaced with nuclease-free water, and the data were expressed in mean ± SD of the three sets of replicated experiments (ns: *p* > 0.05; *p < 0.05; ****p < 0.0001).

Since the activity of CRISPR/Cas12a determines the sensitivity of the RPA-CRISPR method, we investigated its reaction dynamics. We diluted the concentration of the recombinant plasmids to 10^5^ copies/μl as a template to optimize the analytical performance. We first optimized the concentration of crRNA and the ssDNA-reporter and then verified the optimal value of the system’s concentration with different genes of *H. pylori*. In combination, we found that the signal-to-noise (S/N) ratio in with increasing concentration of crRNA, which was maximum at a crRNA different genes detection systems gradually increased concentration of 50 nM, thus reaching the platform period. Conversely, with a further increase in crRNA concentration, the S/N ratio showed a decreasing trend (Figure 3A). Meanwhile, ssDNA-reporter in different genes detection systems have the highest S/N ratio at 1,500 nM (Figure 3B), while the S/N ratio at 2,000 nM was recorded during the platform period. Since the S/N ratio at 500 nM was already sufficient for our detection limits, we decided to set the concentration of the ssDNA-reporter to 500 nM.

To evaluate the specificity of the RPA-CRISPR method, we diluted the concentration of the successful template to 10^5^ copies/μl and used CRISPR/Cas12a to detect different genes. The results show that the detection method can accurately identify the *vacA*, *cagA*, and *16SrDNA* of *H. pylori* (Figures 3C–E), showing significant differences. Therefore, our detection method has good specificity.

To assess the sensitivity of the RPA-CRISPR method, we tested different dilutions concentrations of the template. As shown in Figures 3F–H, the fluorescence signal value of all three genes increased continuously with time. No fluorescence signal was detected in the negative samples within 60 min. The fluorescence values of high copy genes rapidly reached the maximum in a short time. The results showed that the limits of detection for *vacA*, *cagA*, *and 16SrDNA* were 10, 5, and 2 copies/μl, respectively (Figures 3I–N). For PCR, agarose gel electrophoresis showed that the limits of detection for *vacA*, *cagA*, and *16SrDNA* were 100, 10, and 1,000 copies/μl, respectively (Supplementary Figures 1A–C). For qPCR, the results showed that the limits of detection for *vacA*, *cagA*, and *16SrDNA* were 100, 10, and 100 copies/μl, respectively (Supplementary Figures 2A–C). Thus, the sensitivity of the RPA-CRISPR method is higher than that of the conventional molecular detection method.

### Optimization of the Detection Time

**FIGURE 4:**
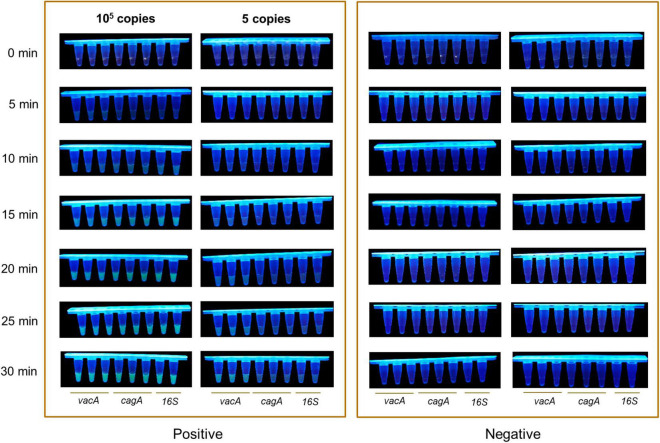
Optimization of the detection time. The visual fluorescence intensity of high and low copy samples detected with the RPA-CRISPR method by Light Cycler 480 fluorescence detection system in 30 min.

Since the detection time is important for point of care products, we optimized the detection time of the RPA-CRISPR method. We increased the concentration of the ssDNA-reporter to 1,000 nM using 10^5^ copies/μl and five copies/μl of plasmids of *vacA*, *cagA*, and *16SrDNA* of *H. pylori* as the positive template and water as the negative control. After RPA, the amplification product was mixed with CRISPR/Cas12a. At the same time, 10 μl of the product was collected to quantify the fluorescence signal in the Light Cycler 480 fluorescence detection system at 37°C. The 21.5 μl of the mixture reacted at 37°C was monitored with the naked eye under UV light and recorded by a camera device every 5 min. The results of the real-time fluorescence signal based on the RPA-CRISPR method showed that in the positive samples of the *vacA*, *cagA*, and *16SrDNA* of *H. pylori*, the fluorescence signal values increased rapidly in the 10^5^ copies/μl and 5 copies/μl samples, and the rate of increase of the fluorescence signal values stabilized at 30 min for the 10^5^ copies/μl samples. The rate of increase of the fluorescence signal was slower for the three genes of 5 copies/μl. In contrast, no fluorescent signal was produced in the negative samples within 60 min (Supplementary Figures 3A–C). The results of visual monitoring based on the RPA-CRISPR method showed that positive samples of *vacA*, *cagA*, and *16SrDNA* with, 10^5^ copies/μl showed strong fluorescence intensity at 10 min, the positive samples of *vacA*, *cagA*, and *16SrDNA* with 5 copies/μl showed observable fluorescence at 20 min and reached the strongest fluorescence intensity at 30 min for both low-copy and high-copy positive samples. In contrast, the fluorescence signal was not observed in negative samples during the whole experiment (Figure 4). After RPA, the CRISPR/cas12a detection method can identify the positive template of *H. pylori* after 10 min incubation at 37°C, with a detection limit as low as a single copy, thereby achieving the goal of *H. pylori* quick detection.

### Establishment and Optimization of One-Tube *Helicobacter pylori* Detection Method Based on RPA-CRISPR Detection Method

**FIGURE 5:**
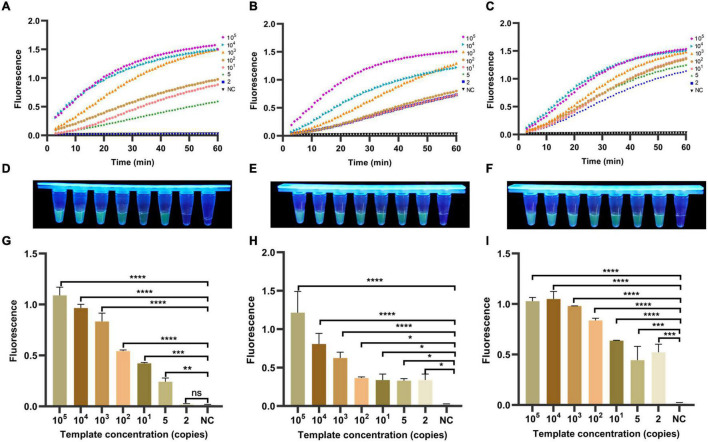
Determination of sensitivity of one-tube RPA-CRISPR method for *H. pylori*. The fluorescence curves of **(A)** *vacA*, **(B)** *cagA*, and **(C)** *16SrDNA* generated by Light Cycler 480 fluorescence detection system at each dilution within 60 min. The fluorescence intensity values with diluted templates of **(D)** *vacA*, **(E)** *cagA*, and **(F)** *16SrDNA* are detected by UV device at 30 min, and the corresponding fluorescence values with diluted templates of **(G)** *vacA*, **(H)** *cagA* and **(I)** *16SrDNA* are quantified by the Light Cycler 480 fluorescence detection system at 30 min. Negative controls (NC) were replaced with nuclease-free water, and the data were expressed in mean ± SD of the three sets of replicated experiments (ns: *p* > 0.05; *p < 0.05; **p < 0.01; ***p < 0.001; ****p < 0.0001).

To ensure an operation and reduce aerosol contamination, we added the RPA products and CRISPR/Cas12a cracking experiments in the same container. As shown in Figures 5A–C, the fluorescence signal value of three genes increased continuously with time. No fluorescence signal was detected in the negative samples within 60 min. Meanwhile, the results of visualized fluorescence signal detection illustrated that the obvious difference in fluorescence intensity could be distinguished with the naked eye between the 10^5^ and 2 copies/μl positive samples (In *cagA* and *16SrDNA*) and the negative samples at 30 min (Figures 5D–F). Differently, the limit of detection in *vacA* is 5 copies/μl. Moreover, Figures 5G–I further illustrates statistically that the sensitivity of our RPA-CRISPR detection method for the three target genes of *H. pylori* is 5, 2, and 2 copies/μl, respectively, at 30 min. In summary, the one-tube RPA-CRISPR rapid detection method showed strong sensitivity, and the limit of detection within 30 min was close to the single-molecule level.

### Design of a Portable Array Detector for Visible Analysis of Thermostatic Nucleic Acid Amplification

**FIGURE 6:**
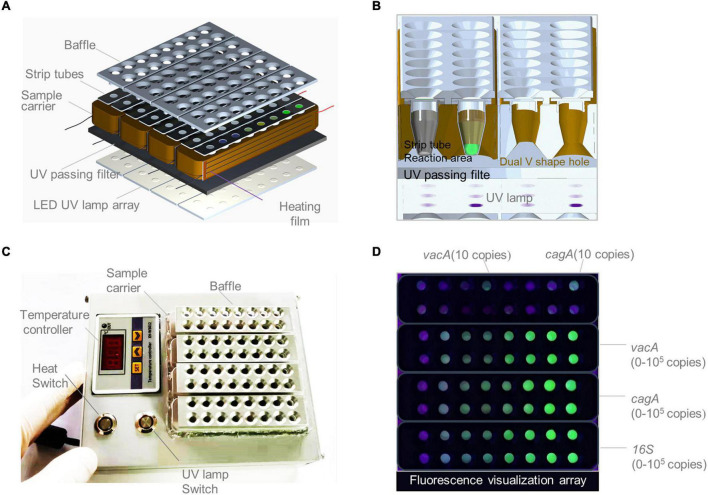
Schematics of a portable array detector (Pad-VATA) for visual analysis of thermostatic nucleic acid amplification and its performance assessment. **(A)** Schematic diagram of the layered structure of the Pad-VATA device, which from up to bottom consists of a baffle plate, a strip tube, a heating film, a UV through filter, and an array of LED UV lamps. **(B)** The operating principle of the Pad-VATA core detection module. **(C)** Physical diagram of Pad-VATA. It is divided into temperature controller module, heating switch module, UV lamp switch module, and decoding function module. The sample was put into the well after the device is powered on, then turn on the heating switch and UV switch to perform the whole sample reaction process. **(D)** The results of different diluted samples detected by the one-tube RPA-CRISPR platform in 20 min.

As shown in Figure 6A, a Pad-VATA was constructed from bottom to top; it was composed of a UV lamp source array, temperature control components, filter plates, and aluminum baffles. The special feature is the inclusion of such a filter plate on the UV lamp. The UV light passing through the filter plate is intended to pass through a portion of the UV spectrum (230–400 nm) while blocking visible light or other unwanted bandwidths and reducing the background. The special design of the aluminum baffle also blocks unnecessary light sources passing through the transparent reaction tube, which further attenuates the background light source and facilitates clear discrimination of the fluorescence signal by naked eyes. As shown in Figure 6B, the sample aperture was designed as a double V-shape with both upper and lower openings to ensure sufficient excitation intensity in the reaction zone at the bottom of the strip tube during UV detection. As shown in Figure 6C, the Pad-VATA was designed as a cuboid box (L × W × H = 150 × 125 × 50 mm, 0.5 kg), which allows the integration of the reaction process and is easily portable. The results of the one-tube RPA-CRISPR-assay reaction on the Pad-VATA for different dilutions of samples at 20 min are shown in Figure 6D. The two positive samples in the first row were *vacA* and *cagA*, each at a concentration of 10 copies/μl, while the remaining wells were *vacA* negative controls; the second row were *cagA* negative controls. The third row from left to right shows the fluorescence reactions of different copy numbers of *vacA* (NC, 2, 5, 10, 10^2^, 10^3^, 10^4^, and 10^5^ copies/μl), and the fourth row was a repeat of the third row. The fifth row from left to right shows the fluorescence reactions of different copy numbers of *cagA* (NC, 2, 5, 10, 10^2^, 10^3^, 10^4^, and 10^5^ copies/μl), and the sixth row was a repeat of the fifth row. The seventh row from left to right was the fluorescence response of *16SrDNA* at different copy numbers (NC, 2, 5, 10, 10^2^, 10^3^, 10^4^, and 10^5^ copies/μl), and the eighth row was a repeat of the seventh row. Nuclease-free water was used as negative controls. These results showed that the fluorescent signal output from the one-tube RPA-CRISPR assay can be accurately identified by naked eyes based on Pad-VATA.

### Detection of Mimicked Clinical Saliva Samples

**FIGURE 7:**
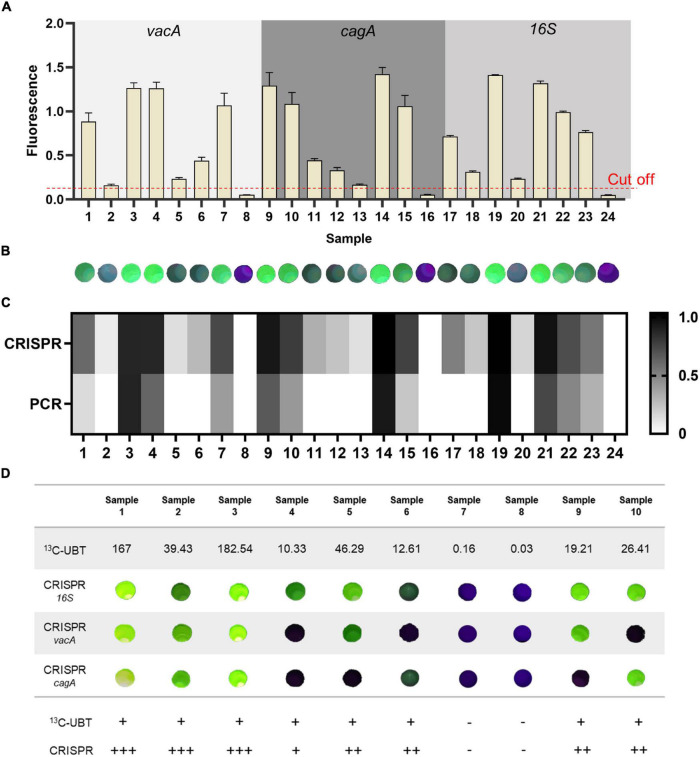
The diagnostic results of the samples by the one-tube RPA-CRISPR platform. The result of the one-tube RPA-CRISPR assay to the mimicked clinical saliva samples based on **(A)** Light Cycler 480 fluorescence detection System and **(B)** Pad-VATA (The dashed line indicates the threshold of the positive result). Results are presented as the mean ± SD, and each sample is repeated three times. **(C)** Comparison results between the one-tube RPA-CRISPR platform for the detection of mimicked clinical saliva samples and qPCR for the detection of different concentrations of plasmids. **(D)** *H. pylori* detection in 10 *H. pylori*-infected clinical samples with proposed one-tube RPA-CRISPR platform and ^13^C-UBT. *H. pylori* positivity was defined based on ^13^C-UBT results. The delta over baseline (DOB) value ≥ 4 is positive. +, indicates a positive sample; -, indicates a negative sample.

To assess the accuracy and reliability of our one-tube RPA-CRISPR platform in mimicked clinical samples of *H. pylori* infection, we tested 21 positive and three negative mimicked clinical saliva samples, and the sample concentration is shown in Supplementary Figure 4A. If the average signal of the *H. pylori* test result is equal to or greater than the negative control sample plus a threshold of 3 times the standard deviation, the sample is considered positive (Kim et al., 2016). According to this standard, the collected saliva samples added to the plasmid were heated and boiled, and *H. pylori* was tested using the one-tube RPA-CRISPR platform. Due to the high requirements of qPCR amplification on the sample, plasmids mixed with saliva cannot be amplified. The diluted plasmids were detected by qPCR. The results showed that the method based on the CRISPR/Cas12a was highly sensitive (Figures 7A,B), compared with the qPCR: eight samples were not detected by qPCR for *H. pylori*, while our CRISPR assay could detect (Figure 7C and Supplementary Figure 4B). Consequently, the one-tube RPA-CRISPR platform has advantages in sensitivity, also in sample handling and detection time to qPCR.

### Detection of Clinical Saliva Samples

To validate the reliability of the one-tube RPA-CRISPR platform, saliva samples from 10 volunteers were collected for testing (the sample serial numbers were the same as the volunteer serial numbers). Volunteers 1–6 were identified as symptomatic infected patients, volunteers 9 and 10 were identified as asymptomatic infected patients, and volunteers 7 and 8 were identified as uninfected after diagnosis by Tang du Hospital. (1) The Pad-VATA can be used to detect *H. pylori* in symptomatic and asymptomatic patients, which is consistent with the ^13^C-UBT and clinical diagnosis of physicians. As shown in Figure 7D and Supplementary Figure 5, among samples detected by Pad-VATA, eight samples (Samples 1–6 and Samples 9 and 10) were positive and two samples (Samples 7 and 8) were negative, which demonstrated our detection platform has good specificity. Moreover, the intensity of the fluorescent signal apparently reflected the degree of infection. Notably, there were multiple mixed genotypes of infection among the 8 *H. pylori* positive samples, of which all *16SrDNA* genes were detected. (2) The detection rates of *vacA* and *cagA* genes were 50 and 62.5%, respectively. (3) The results showed that, in samples 1, 2, and 3, the fluorescence was detectable and stronger in three genes, which indicated that the patient was more severely infected with *H. pylori.* In conclusion, the development of this platform has improved the detection of *H. pylori*, enabling the immediate specific detection of *H. pylori* genes and identification of asymptomatic infected, enhancing the operability of screening in this disease.

## Discussion

*Helicobacter pylori* is highly infectious, and it easily develops into gastritis, even gastric ulcer and gastric cancer when left undiagnosed and untreated. At present, the ^13^C-UBT is still the most widely used and recommended for the non-invasive diagnosis of *H. pylori* infection (Wang et al., 2015). However, the test still has some shortcomings with its performance in epidemiology and evaluation results after treatment. First, the test is prone to false-negative results in patients taking antibiotics and other drugs (Bessede et al., 2017). In addition, the detection accuracy in children (especially for children younger than 6 years old) is only about 70% (Honar et al., 2016; Kotilea et al., 2019). Therefore, a newly rapid, accurate, and cost-effective diagnostic method is urgently needed for effectively preventing and controlling *H. pylori* transmission. For pathogen detection, the introduction of a comfortable self-collection method could help reduce the risk of cross-contamination and increase patient acceptance. In addition to gastric biopsy tissue samples, *H. pylori* can also be taken from some less invasive methods such as stool, oral saliva, or oral dental plaque (Bordin et al., 2021). Among these less invasive methods, saliva has clear advantages for collection and subsequent processing. Currently, saliva-based detection of *H. pylori* is generally divided into two types: one is antigen detection by immunochromatographic assay with a detection rate of 58.89%, which is less accurate (El Khadir et al., 2016), and the other is nucleic acid detection by qPCR, which is gradually being widely used for *H. pylori* detection because of its high accuracy (Momtaz et al., 2012; Wongphutorn et al., 2018). However, qPCR amplification requires highly purified nucleic acids from complex samples. It also requires large advanced equipment and professional personnel for operation, which is not suitable for large-scale on-site epidemiological screening (Pohl et al., 2019; Gong and El-Omar, 2021), limiting the efficiency of detection. Currently, rapid and portable pathogen detection methods based on CRISPR nucleic acid detection systems have been developed and used widely, especially in COVID-19, with satisfied diagnostic performance for both saliva samples and pharyngeal swab samples (Abdollahi et al., 2022). In this study, we detected *H. pylori* in mimicked saliva samples based on the one-tube RPA-CRISPR platform. The results showed that in 21 positive samples, the detection rate of qPCR was only 62%, while the detection rate of the one-tube RPA-CRISPR platform was close to 100%. Second, the qPCR method requires complex nucleic acid extraction from clinical saliva samples, while the one-tube RPA-CRISPR platform does not require this step, and the samples can be tested after rough processing, which greatly simplifies the experimental operation. In summary, the one-tube RPA-CRISPR platform performs well than qPCR in sensitivity and operability. Besides, compared with assays such as endoscopy, the platform does not cause invasive harm to the body.

*Helicobacter pylori* includes many different virulent genotypes, possessing different pathogenic intensities. Clinical studies showed that the main genotypes of *H. pylori* infection include the following: *vacA*, c*agA*, *ureA/B*, and *iceA* (Patel et al., 2014). Genotypes and proportions that varied among populations in different regions were infected with *H. pylori*. A study reported that the detection rate was about 69% for *vacA*, 49% for *cagA*, and 65% for *iceA* in 53 patients from Chile using PCR, among whom about 21% were infected with two or more genes at the same time (Araya et al., 2004). Abu-Taleb et al. (2018) investigated that the positivity rate for *cagA* was about 90% in East Asia (e.g., Japan and Korea) and about 60% in North America, Europe, and Cuba. The differences in the prevalence of *vacA* and *cagA* across regions are mainly attributed to individuals, socioeconomic, geographic, and genetic factors (Abu-Taleb et al., 2018). Studies have shown that patients infected with *H. pylori vacA* and *cagA* are at higher risk of peptic ulcer, gastric atrophy, and gastric cancer than other genotypes (Tanih et al., 2010; Lopes et al., 2014). Similarly, patients with digestive system disorders often have a mixed infection with *vacA* and *cagA*. Therefore, we chose *vacA* and *cagA* to validate the effectiveness of the platform. Interestingly, we found that *H. pylori*-positive patients with high ^13^C-UBT value (>35 in our results) were mostly positive for both *vacA* and *cagA*, while with only one genotype when the value is ≤35. This may indicate that the ^13^C-UBT value reflects the degree of *H. pylori* infection to some extent. This study has clinical directed significance for patients infected with *vacA* and *cagA* genotype, highlighting the importance of prevention. In summary, the experimental results of *H. pylori* infection with *vacA*, *cagA*, and *16SrDNA* genes in clinical samples suggest that the platform we developed improves the accuracy of *H. pylori*-positive patients and detection rate of high-risk type, meanwhile, identifying patients at high risk for developing gastric cancer. However, we have not evaluated patients infected with other *H. pylori* genotypes. We will design a complete testing system in the subsequent study to cover all genotypes of *H. pylori* as many as possible and further investigate the relationship between different *H. pylori* genotypes and the degree of disease.

At present, many CRISPR-based molecular diagnostics are relying on paper-based signal outputs. Although these paper-based methods do not require any large equipment to read the assay results, they have a much lower limit of detection formula than fluorescent signal-based assays (Qiu et al., 2021). In contrast, many CRISPR-based fluorescence output detection platforms still require large blue light transilluminators, which cannot be widely used for point-of-care testing and distributed operations. Moreover, they may produce false-positive test results due to aerosol contamination as the tube’s lid need to be opened several times during the test. To balance the portability and accuracy to meet the needs of practical applications, this article proposed a novel *H. pylori* detection method, which is rapid, easy to operate, adaptable, and highly accurate. The method integrates RPA and CRISPR lysis experiments so that they are integrated into a single reaction step, which greatly reduces the labor and the probability of contaminations caused by tube-opening operations. In addition, the platform constructed in this article does not require specialized operators or detection instruments. The whole reaction process is integrated into our designed portable array detector, which is designed with 64 reaction wells and can detect multiple samples in one step (usually 30 min). In particular, the filter plate on the detector is designed to filter only the UV spectrum (230–400 nm), thereby reducing the background and allowing a clearer fluorescence signal to be read directly by naked eyes. Based on the platform from this study, the proposed platform could detect the conserved gene fragments of *H. pylori* with a concentration as low as two copies/μl. The platform is fast, convenient, efficient, and inexpensive and can be widely used in various settings, such as homes and clinics.

## Data Availability Statement

The original contributions presented in the study are included in the article/Supplementary Material, further inquiries can be directed to the corresponding authors.

## Author Contributions

ZL and LeW conceived and managed the research and analyzed the data. BD, AX, DQ, GC, LiW, WW, and DZ performed the experiments and analyzed the data. BD, AX, ZL, and LeW wrote and reviewed the manuscript. All authors contributed to the article and approved the submitted version.

## Conflict of Interest

The authors declare that the research was conducted in the absence of any commercial or financial relationships that could be construed as a potential conflict of interest.

## Publisher’s Note

All claims expressed in this article are solely those of the authors and do not necessarily represent those of their affiliated organizations, or those of the publisher, the editors and the reviewers. Any product that may be evaluated in this article, or claim that may be made by its manufacturer, is not guaranteed or endorsed by the publisher.

## Footnotes

1
http://bioinfolab.miamioh.edu/CRISPR-DT/interface/Cpf1_main.php
