# Supplementary material for: Rapid and Sensitive Assay of Helicobacter pylori With One-Tube RPA-CRISPR/Cas12 by Portable Array Detector for Visible Analysis of Thermostatic Nucleic Acid Amplification
Source: Front Microbiol. 2022 May 2;13:858247. doi: 10.3389/fmicb.2022.858247 (PMC9108776; doi:10.3389/fmicb.2022.858247)
Supplement: Supplementary file 1 [file Data_Sheet_1.docx]

Supplementary Material

# Supplementary Data

The sequences of inserts in the recombinant plasmids. The *vacA* was indicated in yellow. The *cagA* was indicated in green. The *16S* was indicated in grey.

**Recombinant plasmid 1**

TATAAACTTGTGGTAGTTGGAGCTGATGGCGTAGGCAAGAGTGCCTTGACGATAGGTGATTTTGGTCTAGCTACAGTGAAATCTCGATGGAGTGGGTCCCATTGTCAAGATCACAGATTTTGGGCTGGCCAAACTGCTGGGTGCGGAAAATTCCCGTCGCTATCAAGGAATTAAGAGAAGCAACATCTCCGAAAGCCGAAACAAATGAATGATGCACATCATGGTGGCTGGACAACACACGAGATCCTCTCTCTGAAATCACTGAGCAGGAGATGAAGTGGATATGAAAGACGCTGTAGGGACTTATAAGCTTTCAGGGCTAAGAAACTACACTGGTGGGGATTTAGATGTCAATATGCAAAAAGCCACTTTGCGTTTGGGCCAATTCAATGGCAATTCTTTCACAAGCTTTAAGGATAGCGCTGATCGCACCACGAGAGTGTTTGCTGGCATAATCAATTATTTGTTACTGTGGTAGATACCACTCGCAGTACCAATTTAACAATATGTGCTTCTACACAGTCTCCTGTACCTGGGCAATATGATGCTACCAAATTTAAGCAGTATAGCAGACATGTTGAG

**Recombinant plasmid 2**

TATAAACTTGTGGTAGTTGGAGCTGTTGGCGTAGGCAAGAGTGCCTTGACGATAGGTGATTTTGGTCTAGCTACAGAGAAATCTCGATGGAGTGGGTCCCATTCAAGATCACAGATTTTGGGCGGCCAAACTGCTGGGTGCGGAAAATTCCCGTCGCTATCAAAACATCTCCGAAAGCCGAAACAAATGAATGATGCACGTCATGGTGGCTGGACAACACACGAGATCCTCTCTCTAAAATCACTGAGCAGGAGAAGACTTTATCAATAAGAGCAATGATTTAATCAACAAAGACGCTCTCATTGATGTAGAATCTTCCACAAAGAGCTTTCAGAAATTTGGGGATCAGCGTTACCGAATTTTCACAAGTTGGGTGTCCCATCAAAACGATCCGTCTAAAATCAACACCCGATCGATCCGAAATTTTATGGAACATATCATACAACCCCCTATCCCTGATGACAAAGAAAAAGCAGAGTTACCAGGGTATCTAATCCTGTTTGCTCCCCACGCTTTCGCGCAATCAGCGTCAGTAATGTTCCAGCAGGTCGCCTTCGCAATGAGTATTCCTCTTGATCTCTACGGATTTTACCCCTACACCAAGAATTCCACCTACCTCTCCCACACTCTAGAATAGTAGTTTCAAATGCAGTTCTATGGTTAAGCCATAGGATTTCACACCTGACTGACTATCCCGCCTACGCGCTCTTTACGCAATGGCATTTGTTGGGGTAACCAACTATTTGTTACTGTTGTTGATACTACACGCAGTACAAATATGTCATTATGTGCTGCCATATCTACTTCAGAAACTACATATAAAAATACTAACTTTAAGGAGTACCTACGACATGGGGAGGAATATG

# Supplementary Figures and Tables

## Supplementary Tables

**Table S1:** Detailed sequences of primers, crRNAs, and probes in this study.

| **PCR** | |
| --- | --- |
| ***vacA*-F** | **5'CAGGGCTAAGAAACTACACTGGT3'** |
| ***vacA*-R** | **5'GTGCGATCAGCGCTATCCTT3'** |
| ***cagA*-F** | **5'CAACAAAGACGCTCTCATTGATGT3'** |
| ***cagA*-R** | **5'AATTTCGGATCGATCGGGTG3'** |
| ***16SrDNA*-F** | **5'GGTCGCCTTCGCAATGAGTA3'** |
| ***16SrDNA*-R** | **5'CGTAGGCGGGATAGTCAGTC3'** |
| **RPA** | |
| ***vacA*-F** | **5' TGAAGTGGATATGAAAGACGCTGTAGGGAC 3'** |
| ***vacA*-R** | **5' TTGCCATTGAATTGGCCCAAACGCAAAGTG 3'** |
| ***cagA*-F** | **5' CAAAGACGCTCTCATTGATGTAGAATCTTCCAC 3'** |
| ***cagA*-R** | **5' ACTCTGCTTTTTCTTTGTCATCAGGGATAGGG 3'** |
| ***16SrDNA* -F** | **5' CCCACGCTTTCGCGCAATCAGCGTCAGTAATGTTCC 3’** |
| ***16SrDNA* -R** | **5' GCCAGCAGCCGCGGTAATACGGAGGGTGCAAGCG 3'** |
| **crRNA** | |
| ***vacA*** | **5' UAAUUUCUACUAAGUGUAGAUGAUGUCAAUAUGCAAAAAGC3'** |
| ***cagA*** | **5' UAAUUUCUACUAAGUGUAGAUAUGGGACACCCAACUUGUGA3'** |
| ***16SrDNA*** | **5'UAAUUUCUACUAAGUGUAGAUAAAUGCAGUUCUAUGGUUAA 3'** |
| **ssDNA-reporter** | |
|  | **6-FAM-TTATT-BHQ1** |

##
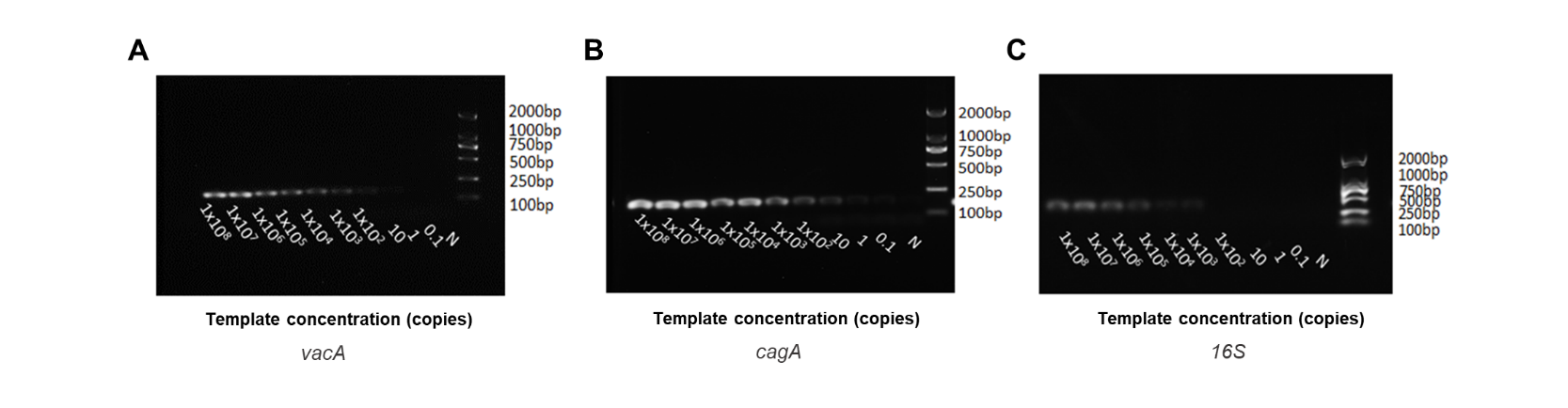
Supplementary Figures


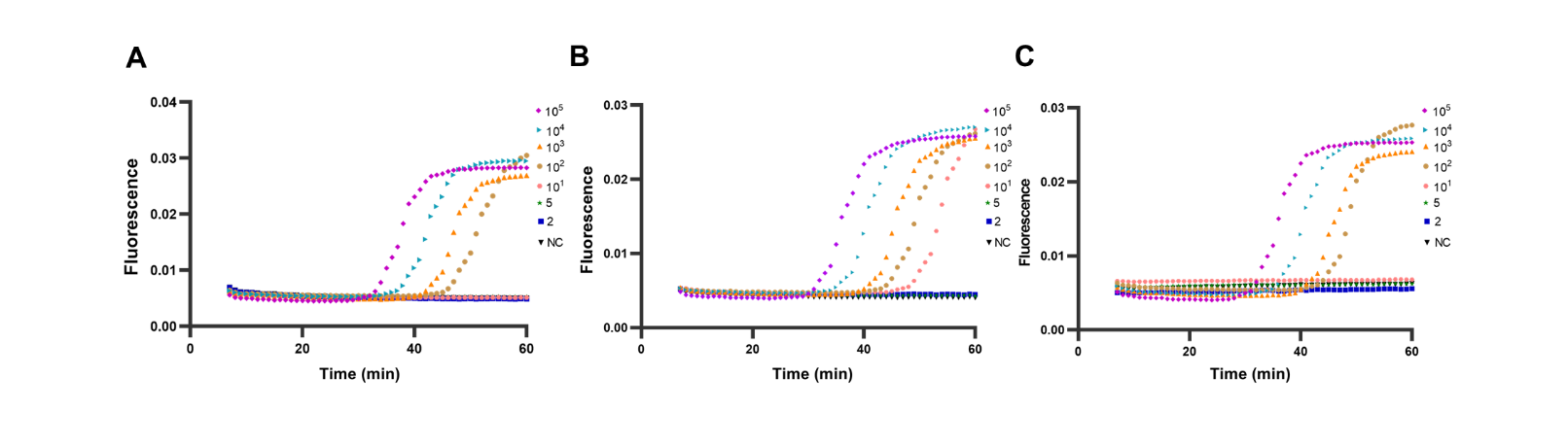
**Supplementary Figure 1.** Analysis of PCR amplification products by agarose gel electrophoresis. PCR amplification with 10-fold gradually diluted DNA as templates. 10 μL of amplified products are gel electrophoresed and **(A)** *vacA* **(B)** *cagA* **(C)** *16SrDNA i*maged shown in the bottom.


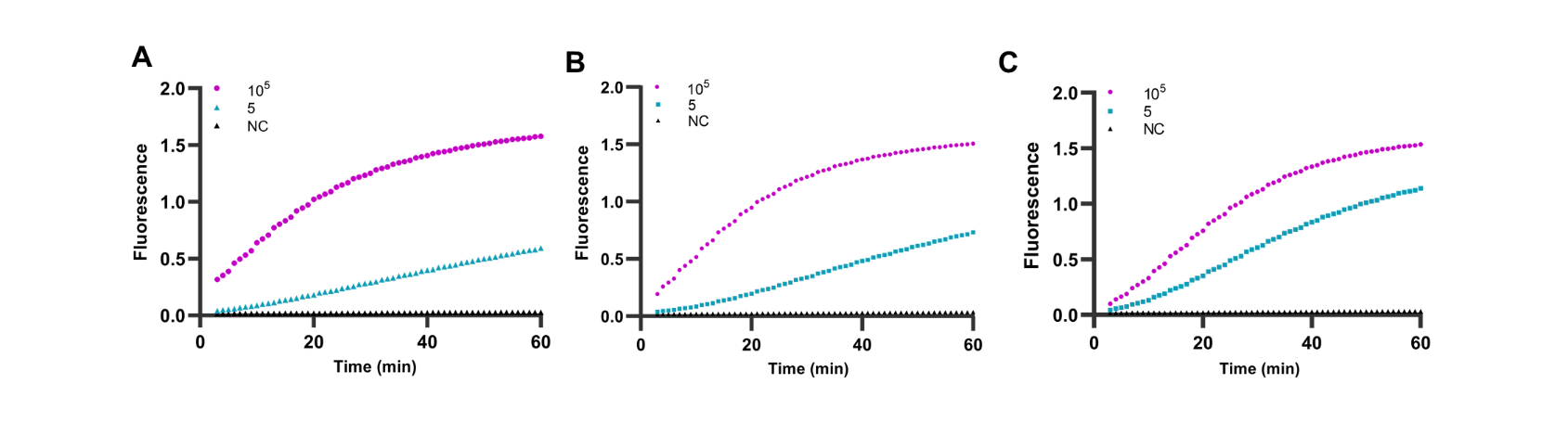
**Supplementary Figure 2.** Real-time PCR with 10-fold gradually diluted DNA as templates. For each template **(A)** *vacA* **(B)** *cagA* **(C)** *16SrDNA* concentration, three repeats are tested simultaneously and one fluorescence curve for each template concentration is shown here.

**Supplementary Figure 3.** Diluted 10^5^ copies/µL and 5 copies/µL of **(A)** *vacA*, **(B)** *cagA* and **(C)** *16SrDNA* when RPA-CRISPR reaction produces fluorescent curves. The data is represented ± SD in hom mean. Three tests in one form. Negative controls (NC)use nuclease-free water as input.

**
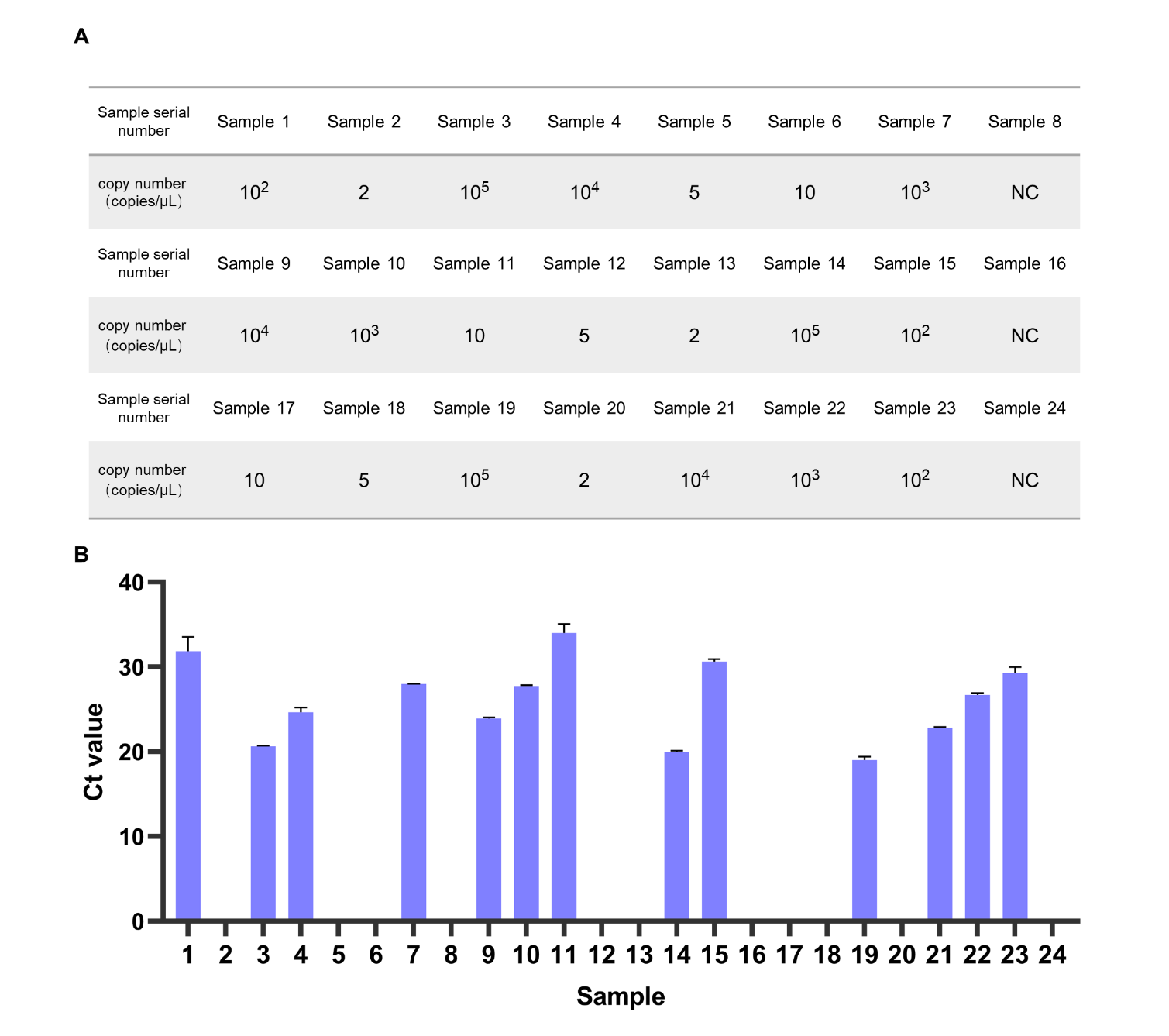
**

**Supplementary Figure 4 (A)** Concentration of plasmids in 24 mimical clinical samples. **(B)** The Ct value of Real time- PCR for the gradient diluted template. Negative control (NC)use nuclease-free water as input. Results depict the mean ±SD of three experimental replicates.


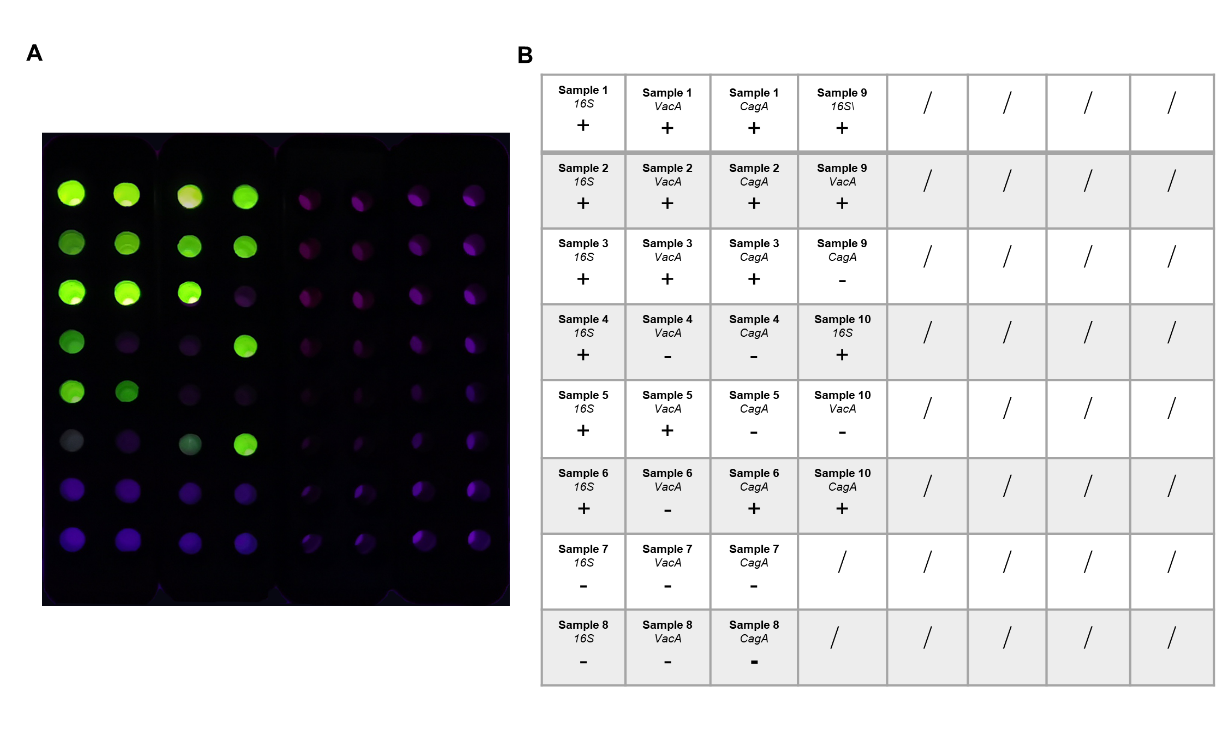


**Supplementary Figure 5.** The results in 10 clinical samples based on one-tube RPA-CRISPR platform for *H. pylori* infection. **(A)** Visualization of fluorescence results based on Pad-VATA. **(B)** Information of the samples and the results of the detection of *H. pylori* genes.
